# SWI/SNF-Related Matrix-Associated Actin-Dependent Regulator of Chromatin Subfamily B Member 1 (SMARCB1)-Deficient Tumor in the Parapharyngeal Space: A Case Report

**DOI:** 10.7759/cureus.69171

**Published:** 2024-09-11

**Authors:** Seiichiro Kamimura, Eiji Kondo, Takahiro Azuma, Go Sato, Yoshiaki Kitamura

**Affiliations:** 1 Otolaryngology - Head and Neck Surgery, Tokushima University, Tokushima, JPN

**Keywords:** ini1, parapharyngeal tumor, rare head and neck, smarcb1, smarcb1/ini1-deficient tumor, swi/snf complex

## Abstract

SWI/SNF-related matrix-associated actin-dependent regulator of chromatin subfamily B member 1 (SMARCB1) is a tumor suppressor gene, and SMARCB1 deficits have been associated with numerous malignant tumors. Carcinomas with a SMARCB1 deficit generally have a poor prognosis. In the head and neck region, there have been many recent cases of SMARCB1-deficient sinonasal carcinoma, but reports of extrasinonasal sites are extremely rare. This study reports a case of SMARCB1-deficient tumor in the right parapharyngeal space. A 64-year-old woman with right neck swelling was presented to our hospital, and imaging revealed a tumor in the right parapharyngeal space. After four years of follow-up, the tumor had increased in size, and a biopsy revealed deficits in SMARCB1 in the tumor cells. No definitive histological diagnoses were made. Radiation reduced the size of the tumor, and the patient survived without progression, three years after completion of treatment.

## Introduction

SWI/SNF-related matrix-associated actin-dependent regulator of chromatin subfamily B member 1 (SMARCB1/INI1) is a core subunit of the SWI/SNF chromatin remodeling complex and a tumor suppressor gene [1]. Deficits in SMARCB1 affect carcinogenesis and tumor growth.

SMARCB1-deficient tumors were first identified in malignant rhabdoid tumors and soft tissue tumors with poor prognoses that occur primarily in children [2]. However, it is now known that epithelial tumors, except soft tissue tumors, also exhibit deficits in SMARCB1 [3]. Reports published in 2014 concerning the head and neck region show that SMARCB1 deficits can be found in sinonasal carcinoma [4-7]; SMARCB1-deficient sinonasal carcinoma was newly described in the 2017 WHO classification of tumors in the nose and sinus region [8].

SMARCB1-deficient sinonasal carcinoma is often diagnosed as an undifferentiated or poorly differentiated carcinoma, with pathological evidence of rhabdoid and basaloid cells [9]. Although the number of cases of SMARCB1-deficient sinonasal carcinoma has been increasing in recent years, reports of SMARCB1-deficient tumors in the head and neck regions, except the nose and sinuses, are extremely rare.

Treatment of SMARCB1-deficient sinonasal carcinoma often involves multimodality therapy such as surgical resection or chemoradiotherapy [7,10]. However, the prognosis is generally considered poor. Due to the limited response to radiation therapy, radiation alone is rarely chosen as a treatment option [10]. Chemotherapy based on cisplatin is sometimes administered, but its efficacy remains unclear. Furthermore, the effectiveness of immune checkpoint inhibitors is suboptimal [10].

Deficit in SMARCB1 gene product alters the function of the SWI/SNF complex, leading to increased zesta homolog 2 (EZH2) activity. As a result, oncogenic pathways such as MYC, sonic hedgehog, and WNT/β-catenin are upregulated, while the transcription of tumor suppressor genes is suppressed. Tazemetostat, a potent EZH2 inhibitor, has emerged as a promising targeted therapy for SMARCB1-deficient malignancies [11]. However, the effectiveness of tazemetostat in SMARCB1-deficient sinonasal tumors, a type of SMARCB1-deficient tumor in the head and neck region, remains unclear [11,12].

Herein, we report a case of SMARCB1-deficient tumor arising in the parapharyngeal space, treated with radiation alone, with a good sensitivity and response to the radiotherapy. To the best of our knowledge, this is the first reported case of a SMARCB1-deficient tumor in the parapharyngeal space.

## Case presentation

**Figure 1:**
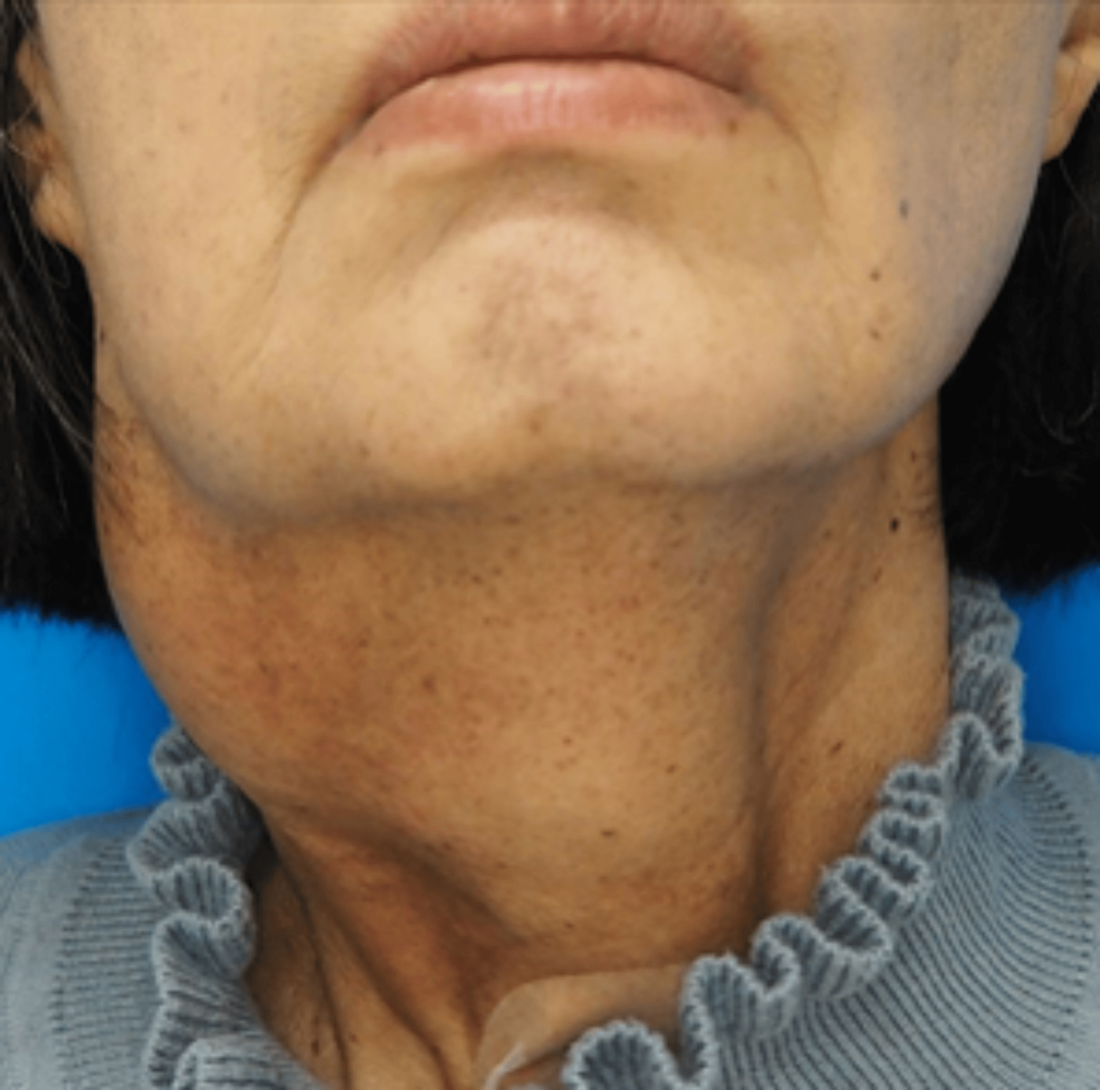
Imaging of the patient’s neck showing a 7 cm mass in the right neck.

A 64-year-old woman visited our hospital with a one-year history of right neck swelling. Contrast-enhanced CT tomography showed a 4 cm mass in the right parapharyngeal space. Suspecting schwannoma or pleomorphic adenoma, we considered surgical excision; however, the mass developed in contact with the carotid artery. The patient declined a blood transfusion, and we decided that safe surgery would be difficult without consent for a blood transfusion; therefore, we opted to wait and scan without treatment. However, the mass grew slowly to 7 cm four years after the initial visit (Figure 1).

**Figure 2:**
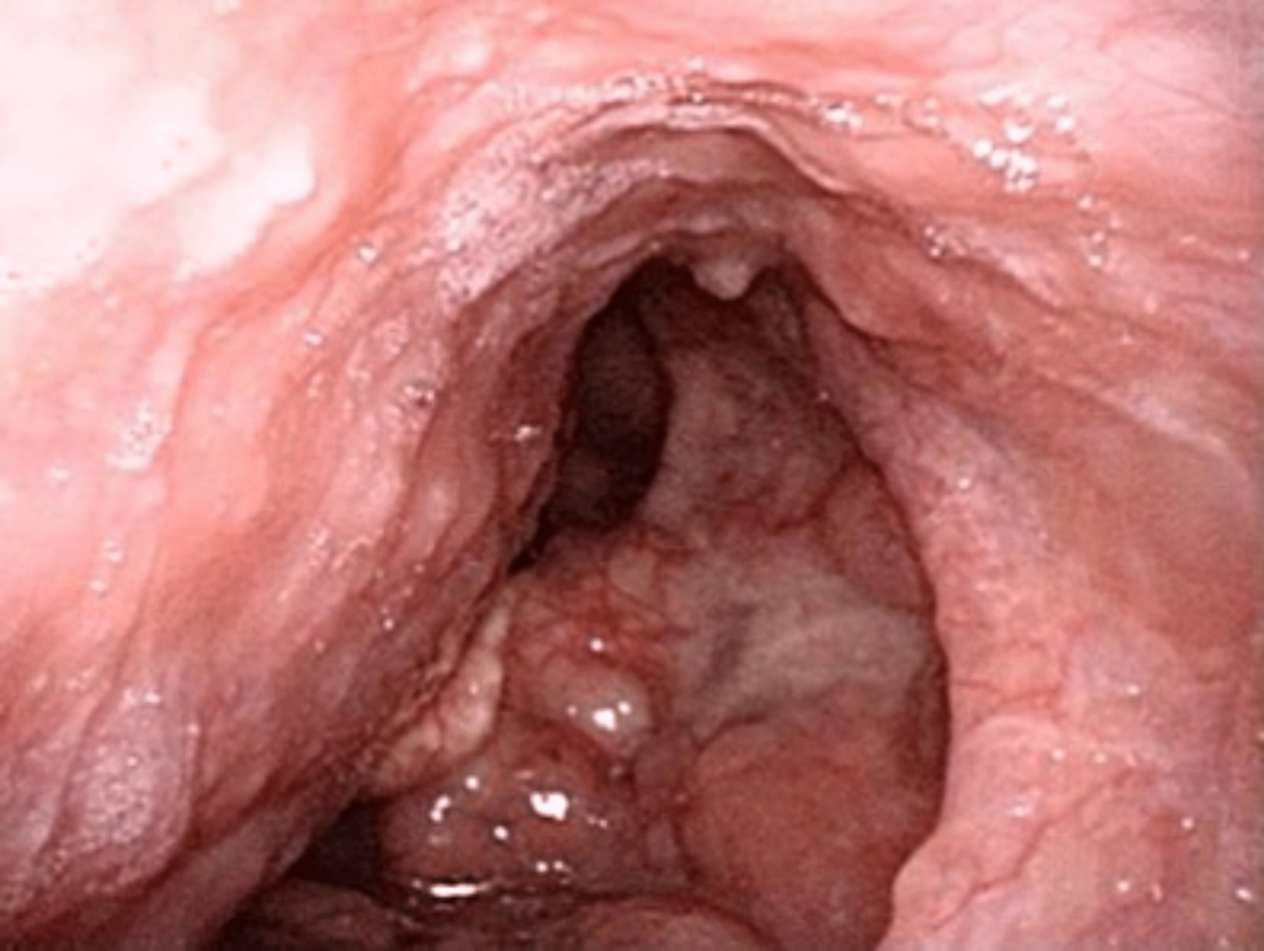
Laryngeal endoscopic image The right side of the oropharynx protrudes, and the glottis cannot be observed.

The patient did not experience any pain or neurological symptoms. Endoscopy revealed a mass protruding from the submucosa to the right side of the parapharynx (Figure 2).

**Figure 3:**
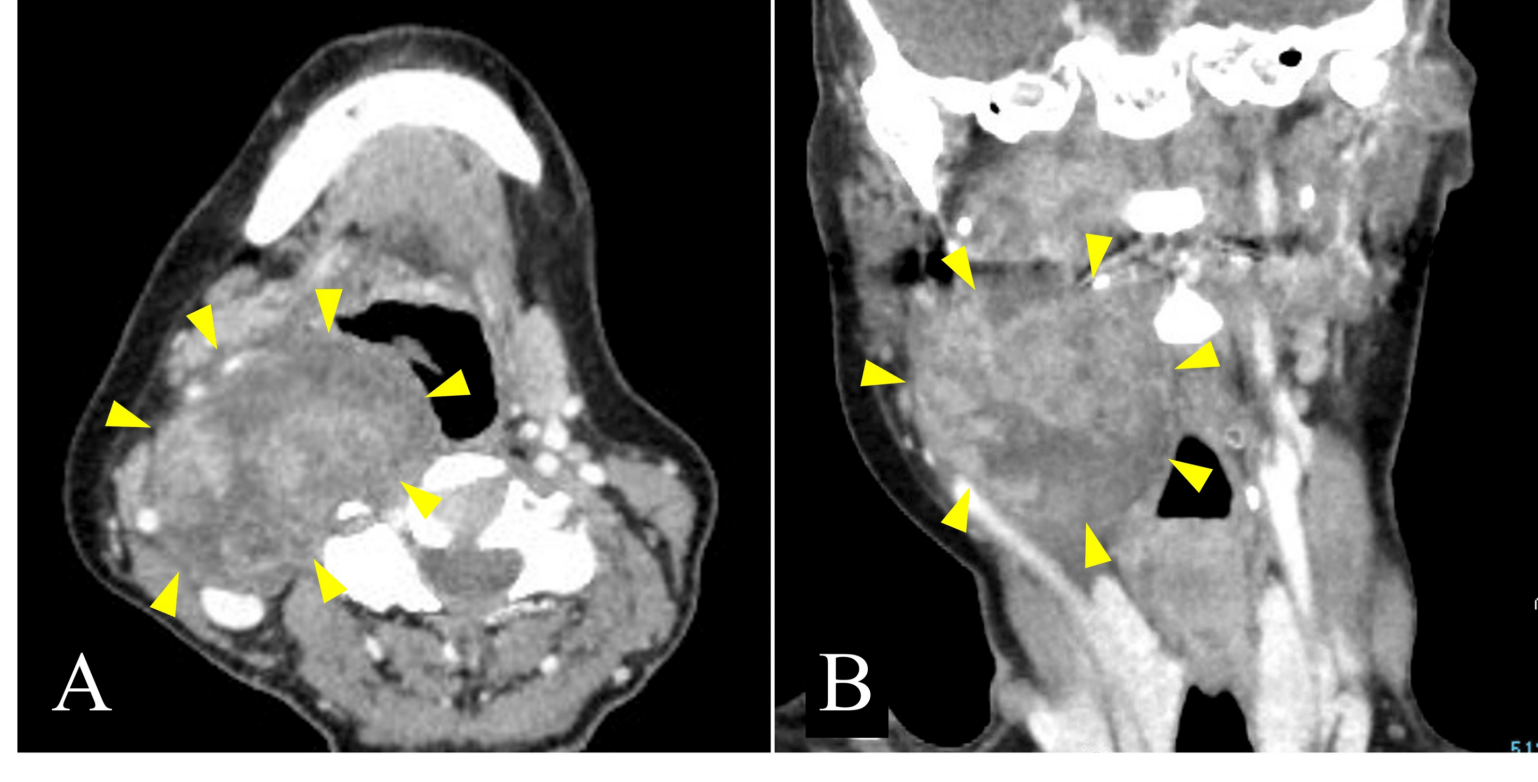
Contrast-enhanced CT image: (A) axial section; (B) coronal section. A mass with a maximum transverse diameter of 7 cm is seen in the right parapharyngeal space. The tumor is heterogeneously contrasted. The internal carotid artery and internal jugular vein are compressed laterally.

Contrast-enhanced CT tomography revealed a heterogeneously contrasting mass with a maximum transverse diameter of 7 cm in the right parapharyngeal space, compressing the internal carotid artery and internal jugular vein laterally (Figure 3).

**Figure 4:**
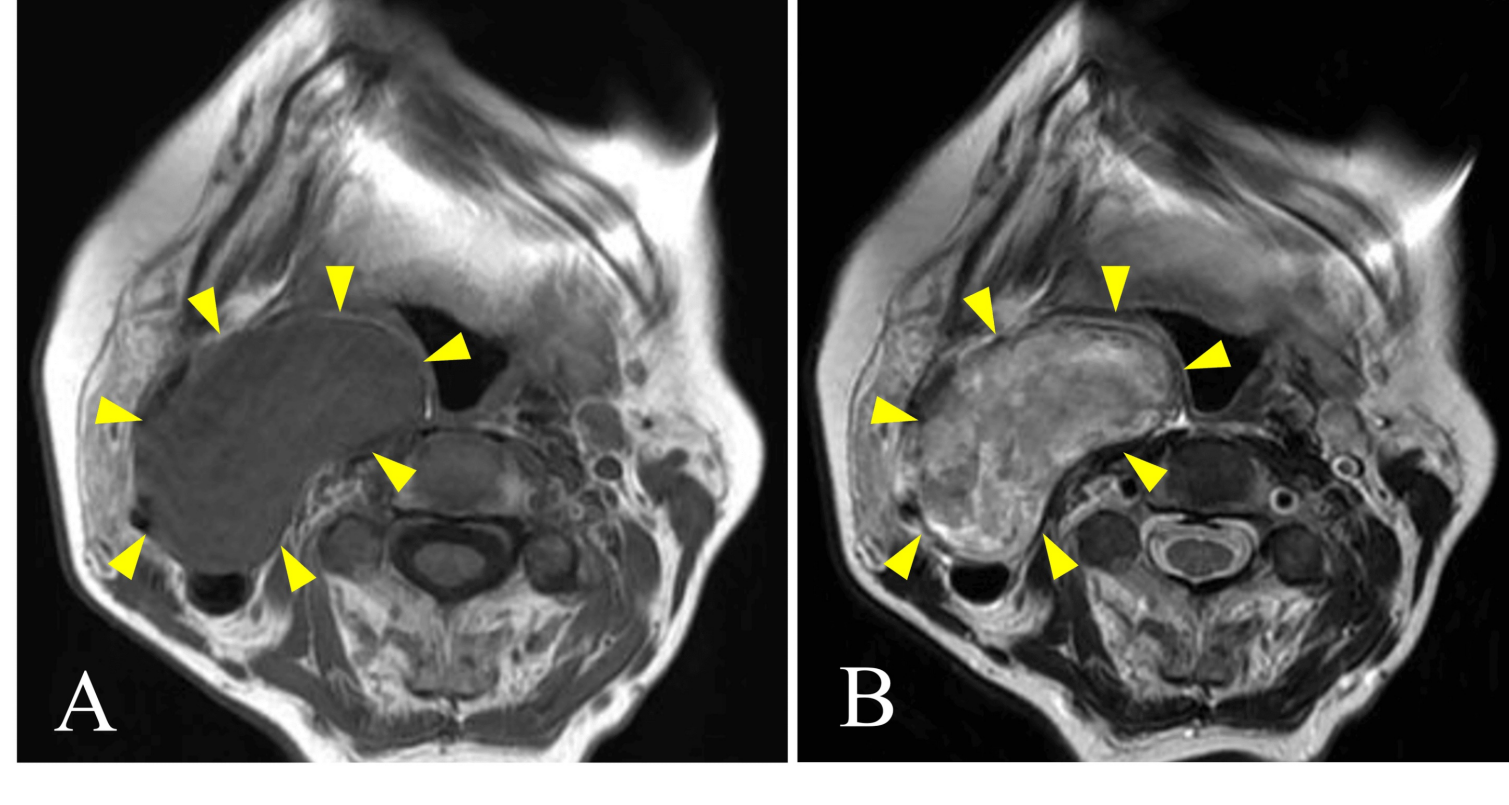
MRI images: (A) T1-weighted image; (B) T2-weighted image. A mass with isointensity on the T1-weighted image and heterogeneous high intensity on the T2-weighted image was seen in the right parapharyngeal space. The border was clear.

MRI showed isointensity on T1-weighted images and heterogeneous high intensity on T2-weighted images, with clear boundaries (Figure 4).

**Figure 5:**
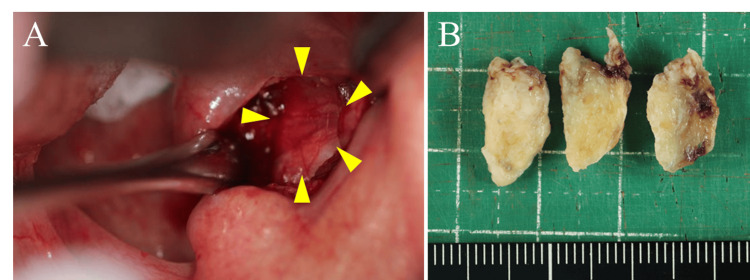
Intraoperative findings and collected tissue: (A) intraoperative findings; (B) collected tissue. The right side of the oropharynx was incised, and a portion of a well-defined submucosal mass (surrounded by arrowheads) was partially resected at a size of 16 mm x 10 mm x 8 mm.

A tracheotomy was performed to maintain the airway, and a transoral biopsy was performed simultaneously. The right side of the oropharynx was incised, and a portion of the well-defined submucosal mass was partially resected to a size of 16 × 10 × 8 mm (Figure 5).

**Figure 6:**
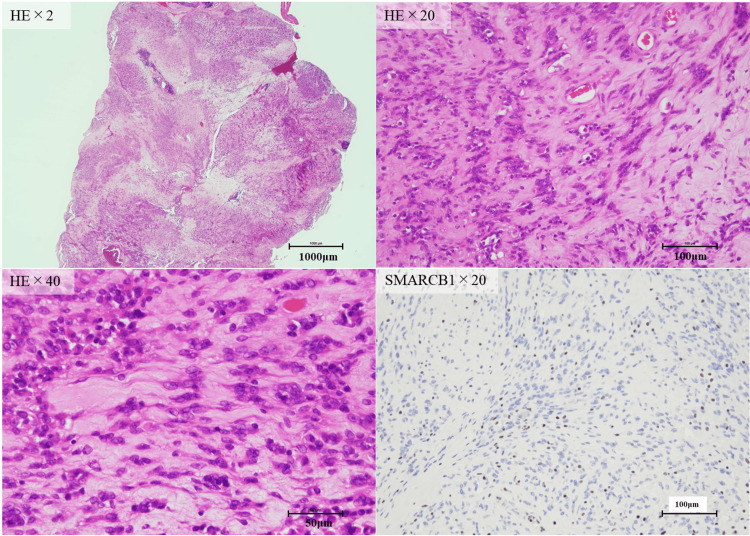
H&E staining and immunostaining for SMARCB1/INI1 Infiltrative growth of round to short spindle-shaped epithelial-like cells was observed. Tumor cells were distributed in small clusters, bundles, and solitary, with some areas of intercellular cleavage. Tumor cells had distinct nucleoli and similarly round nuclei, and the cytoplasm was narrow and weakly acidophilic. Mitosis was seen, but no necrosis was observed. Rhabdoid cells were not observed. Tumor cell nuclei were negative for SNARCB1. SNARCB1: SWI/SNF-related matrix-associated actin-dependent regulator of chromatin subfamily B member 1

Hematoxylin and eosin (H&E) staining showed the infiltrative growth of round to short spindle-shaped epithelial-like cells (Figure 6).

**Figure 7:**
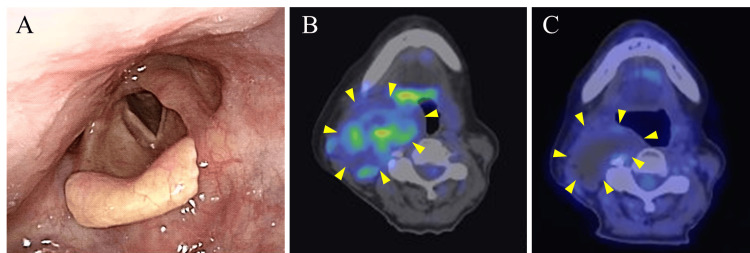
(A) Laryngeal endoscopic image three years after completion of radiotherapy. Protrusion of the right side of the oropharynx was reduced. (B) FDG-PET/CT scan before treatment. (C) FDG-PET/CT scan three years after the end of treatment. Three years after the end of treatment, the tumor had reduced, and FDG accumulation in the tumor was no longer observed. FDG-PET/CT: fluorodeoxyglucose-positron emission tomography/computed tomography

The tumor cells were distributed in small clusters, bundles, and solitary structures, with some areas of intercellular cleavage. The tumor cells had distinct nucleoli and similarly round nuclei, and the cytoplasm was narrow and weakly acidophilic. Mitosis was observed, but no necrosis was observed. No rhabdoid cells were observed in this study. Ki-67 index was 14%. Immunostaining showed that the tumor cells were vimentin-positive but negative for S-100 protein, SOX10, Cytokeratin (AE1/3), desmin, myogenin, MyoD1, smooth muscle actin, SSX, SS18-SSX, CD34, and ERG. SMARCB1/INI1 expression was deficient in the nuclei of tumor cells (Figure 6). Nerve sheath tumors, rhabdomyosarcoma, synovial sarcoma, vascular tumors, myoepithelioma, and melanoma were considered in the differential diagnosis, but the immunostaining results did not match any of them. Although a definitive histological diagnosis was not made, we diagnosed a malignant tumor with SMARCB1/INI1-deficient. Oncopanel examination (FoundationOne® CDx; Foundation Medicine, Cambridge, USA) of excised tissue samples also confirmed deficits in SMARCB1. Fluorodeoxyglucose (FDG)-positron emission tomography (PET)-CT showed partial FDG accumulation in the tumor in the right parapharyngeal space but no accumulation in other organs, suggesting no metastasis. Radiation was chosen as the method of treatment, and the tumor was irradiated with 60 Gy/30 Fr of intensity-modulated radiation. The tumor remained but reduced significantly; FDG accumulation on the FDG-PET/CT scan was no longer seen and remained reduced three years after the completion of radiation (Figure 7).

## Discussion

SMARCB1 is one of the core subunits of the SWI/SNF complex, a chromatin remodeling factor complex encoded by INI1/hSNF5/SMARCB1/BAF47 on chromosome 22q11.2. SMARCB1 is widely expressed in normal cells in the nucleus. In humans, there are two types of SWI/SNF complexes, and a relationship between genetic abnormalities in these SWI/SNF complexes and tumor development has been reported [1].

In 1990, a deficit in the long arm of chromosome 22 was reported in malignant rhabdoid tumors, and mutations in SMARCB1 were identified in 1998 [2]. Subsequently, a deficit in SMARCB1 is associated with many malignancies [1]. In head and neck cancer, SMARCB1-deficient sinonasal carcinoma was first reported in 2014 [4,5], followed by a series of reports. Reports indicate that SMARCB1 deficits are found in 2.7% to 6% of sinonasal carcinomas [4,6]. Still, sinonasal tumors account for only approximately 8% of all head and neck tumors, and SMARCB1-deficient tumors in head and neck cancer are rare [13].

Reports on SMARCB1-deficient tumors of the head and neck, except the sinonasal region, are rare. Kezlarian et al. reported three cases of thyroid cancer, two cases of skin cancer, and one case of a lymph node of unknown primary origin [14]. Neves-Silva et al. retrospectively investigated the deficit of SMARCB1 in 643 cases of squamous cell carcinoma of the head and neck except the sinonasal region and reported that a deficit of SMARCB1 was found in four cases, and the onset sites were the lower lip, soft palate, hypopharynx, and vocal cords [15]. Following this report, we estimated that the rate of SMARCB1 deficits in extrasinonasal squamous cell carcinomas in the head and neck region is approximately 0.6%.

There is no standard treatment for head and neck cancer with SMARCB1-deficient tumors, and multidisciplinary treatments, such as surgery, cisplatin-based chemotherapy, and radiation, have been performed [2,10]. However, SMARCB1-deficient tumors in the extrasinonasal region have poor prognoses [15]. Human papillomavirus (HPV)-positive cases have been reported to have a better prognosis than HPV-negative cases, and Epstein-Barr virus (EBV) infection is considered less relevant [15]. In a study analyzing 39 cases of SMARCB1-deficient sinonasal carcinoma, 22 patients were most frequently selected for surgery and chemoradiation, with a disease-free survival rate of 30% during the 11-115 months of follow-up. A total of 11 cases of distant metastasis and 10 cases of recurrence were observed, with three cases of recurrence in the cervical lymph nodes. A total of 17 patients died of SMARCB1-deficient sinonasal carcinoma [7].

Although our patient had a SMARCB1-deficient tumor in the parapharyngeal space, the progression was slow, and the prognosis after radiotherapy was favorable, indicating a rare course. Tumors with SMARCB1 deficiency in the head and neck region are particularly rare [13], with occurrences outside the sinonasal area being extremely uncommon. In this instance, the tumor in the parapharyngeal space showed a favorable response to radiotherapy, resulting in three years of progression-free survival. Such a positive outcome is unusual for SMARCB1-deficient tumors, which generally exhibit resistance to treatment, and is noteworthy.

Recently, the efficacy of tazemetostat, an EZH2 enzyme inhibitor, as a specific treatment for SMARCB1-deficient epithelial tumors, has been reported in some SMARCB1-deficient tumors in adults and children [16]. Dysfunction of the SWI/SNF complex caused by SMARCB1 deficits leads to the release of inhibition of EZH2 activity, which leads to carcinogenesis and tumor growth. Tazemetostat exerts its effect by inhibiting the EZH2 enzyme [17]. In 2020, a phase II trial for progressive epithelioid sarcoma with a SMARCB1 deficit was conducted and was shown to be effective [18]. However, studies on the effect of malignant rhabdoid tumors and synovial sarcoma with a deficit of SMARCB1 were limited [17]. The advent of specific therapies for tumors with deficits in SMARCB1, such as EZH2 enzyme inhibitors, will increase the importance of assessing the presence of deficits in SMARCB1.

## Conclusions

In this study, we reported a rare case of a SMARCB1-deficient tumor in the parapharyngeal space. The tumor reduced in size after radiotherapy and was maintained for three years without regrowth. Specific treatments for SMARCB1-deficient tumors are now available for several carcinomas, and evaluating SMARCB1 deficits will become increasingly important.
